# Phonological awareness mediates the relationship between *DCDC2* and reading performance with home environment

**DOI:** 10.1038/s41539-024-00247-5

**Published:** 2024-05-03

**Authors:** Miao Li, Mellissa M. C. DeMille, Maureen W. Lovett, Joan Bosson-Heenan, Jan C. Frijters, Jeffrey R. Gruen, the GRaD Study Consortium, Richard Boada, Stephanie Gottwald, Dina Hill, Lisa A. Jacobson, Erik G. Willcutt, Maryanne Wolf

**Affiliations:** 1 Department of Curriculum and Instruction, College of Education, University of Houston, Houston, TX USA; 2 Departments of Pediatrics and Genetics, Yale University School of Medicine, New Haven, CT USA; 3 Neurosciences and Mental Health Program, The Hospital for Sick Children, University of Toronto, Toronto, ON Canada; 4 Department of Child and Youth Studies, Faculty of Social Sciences, Brock University, St. Catharines, ON Canada; 5 University of Colorado Denver, Aurora, CO USA; 6 Tufts University, Medford, MA USA; 7 University of New Mexico, Albuquerque, NM USA; 8 Kennedy Krieger Institute and Johns Hopkins University School of Medicine, Baltimore, MD USA; 9 University of Colorado Boulder, Boulder, CO USA; 10 University of California at Los Angeles, Los Angeles, CA USA

**Keywords:** Social sciences, Education

## Abstract

Proficient reading requires critical phonological processing skill that interacts with both genetic and environmental factors. However, the precise nature of the relationships between phonological processing and genetic and environmental factors are poorly understood. We analyzed data from the Genes, Reading and Dyslexia (GRaD) Study on 1419 children ages 8–15 years from African-American and Hispanic-American family backgrounds living in North America. The analyses showed that phonological awareness mediated the relationship between *DCDC2*-READ1 and reading outcomes when parental education and socioeconomic status was low. The association between READ1 and reading performance is complex, whereby mediation by phonological awareness was significantly moderated by both parental education and socioeconomic status. These results show the importance of home environment and phonological skills when determining associations between READ1 and reading outcomes. This will be an important consideration in the development of genetic screening for risk of reading disability.

## Introduction

Proficient reading is critical for success in school as well as lifetime earning potential. Children with low reading ability are more likely to live in poverty and have higher rates of unemployment as adults^1^. A great deal of research has been devoted to investigating the predictors of reading outcomes, including measures of individual word reading and reading comprehension. In English, an opaque orthography, the consensus among reading researchers is that phonological processing skill, particularly phonological awareness, is a significant determinant of English reading outcome^2,3^. Phonological processing refers to the use of phonological information in decoding written language^4^. Children with advanced English phonological skills tend to have successful English reading outcomes, whereas lower phonological skill is associated with reading difficulties^5,6^.

In addition to phonological skills, reading is also influenced by genetic factors. In studies of twins, the heritability of reading is high, ranging from 0.46 to 0.72^7,8^. However, heritability estimates are not uniformly high in molecular genetic studies of unrelated individuals assessed with single nucleotide polymorphisms (SNPs). While the differences in heritability estimates between family studies and SNP studies in psychiatric disorders is well known – frequently characterized as “missing heritability” – it is also likely that the relationship between genes and reading is indirect. Few studies have rigorously addressed the connection between genes and reading outcomes. The prominent role of phonological skill in reading performance suggests that it may function as a mediator between specific genes and reading proficiency.

Approximately 18 genes have been associated with reading performance, but association with only 8 genes has been replicated three or more times: *CMIP*, *ATP2C2*, *FOXP2*, *ROBO1*, *DYXC1*, *KIAA0319*, *DCDC2*, and *CNTNAP2*^9^. Among them, only *KIAA0319* and *DCDC2* are located in the most replicated reading locus (DYX2; chromosome 6p22), and within both genes the peaks of association lie within regulatory features. It has been previously shown that children could be identified with a specific phonological deficit with variants of READ1, a regulatory element encoded within a known RD risk gene called *DCDC2*^10,11^.

Home environment is another factor related to reading, particularly in North American context, by influencing the relationship between genetics and reading. The variance due to genetic influence fluctuates because home environment moderates genetic influences on reading outcomes^12^. Home environment may function as a condition on the mediating effect of phonological skill in the gene-reading linkage. In the present study, we aimed to examine the mediating role of phonological awareness between genes and reading. We were also interested in the moderating role of home environment to influence this mediation effect. By simultaneously considering the roles of phonological awareness and home environment, a moderated mediation model was tested to provide guidance in understanding how genetics affect reading performance.

It is well established that developmental and individual differences in phonological processing are causally related to reading ability in both longitudinal and experimental research^2,13^. Furthermore, deficit in phonological processing is a contributor to reading disability^14,15^.

Phonological awareness, a major component of phonological processing, refers to the sensitivity and ability to manipulate sounds or sound structures of words. It is a powerful concurrent and longitudinal predictor of reading development^3,4,16^. According to the phonological deficit hypothesis, phonological awareness is a critical factor explaining difficulties in reading^17^. Interventions on phonological awareness training are effective in improving reading performance of children with reading disability^5^. Thus, the connections between phonological awareness and reading outcomes are well established and evidenced.

Studies reporting significant and substantial genetic influences on reading performance have shown that reading problems tend to run in families^18,19^. Findings from twin studies have also demonstrated large genetic influences on both word reading and reading comprehension in samples from Colorado, Ohio, Florida, England, Australia, and Scandinavia^7,20–23^. Although behavioral genetics studies can tell us whether reading is affected by genetic influences, they do not tell us which risk gene(s) can influence reading. This issue points to the high need of molecular genetics research.

Patterns of heritability and molecular genetics studies have identified risk genes that may cause reading difficulties^10,24–27^. *DCDC2* and *KIAA3019*, encoded next to each other on chromosome 6p22, are the most replicated risk genes for reading^10,11^. READ1 (regulatory element associated with dyslexia 1) is a regulatory element encoded in intron 2 of *DCDC2* and is a highly polymorphic complex tandem repeat with at least 40 alleles^10,11,28,29^. Among these alleles, RU2-Short, which includes 6 or fewer copies of repeat unit 2 (alleles 4, 10, and 16 etc.), confers significant risk for reading difficulties^30^. Clinical studies have shown READ1 allele-specific association with severe reading and language impairment^29^. Regardless, the total genetic effect summed over the entire genome accounts for only a small portion of variance in reading difficulties^31^. Therefore, READ1 in the DYX2 locus should be further studied for its effects on reading performance.

Previous studies have consistently found a genetic influence on phonological awareness^8,32,33^ as well as interactions between phonological awareness and reading-related outcomes. For example, genetic influences were found to explain the comorbidity among phonological and orthographic skills^8^ and the covariance between phonological awareness, rapid naming, and reading outcomes^33^.

Home environment is crucial in literacy development. Parental education and socioeconomic status (SES) are important indicators of home environment^34–38^. Parent education is considered one of the most stable variables as it is usually established early in life and does not change over time. Parental education is highly correlated with children’s reading achievement^39,40^. SES is typically the most direct measure of family wealth and research studies including meta-analysis have demonstrated that SES is highly correlated with student achievement in North America^40,41^.

Two models have been proposed to understand the relationship between genetic and environmental influences (G × E) on reading. One is the bioecological model which proposes that genetic influences should be greater in advantaged/supportive environments because genetic potential would be more fully realized in advantaged/supportive environments than in disadvantaged/poorly supportive environments^42^. The other is the diathesis-stress model which suggests that heritability should be greater in disadvantaged environments because deleterious genes may not be observed in more advantaged environments. Both models are reasonable accounts of G × E interactions on reading. For example, individuals who carry deleterious-acting variants of genes that raise the risk for reading disability may be exposed to a disadvantaged environment that triggers activation. Conversely, individuals who carry protective alleles may be exposed to a supportive environment that triggers activation.

G × E interactions have been examined in reading abilities and disabilities^1,12,20,43^ but the findings are mixed. For example, Kremen et al.^43^ found a shared environment × parental education interaction but not genetic × parental education interaction in a sample of middle-aged men. Taylor and Schatschneider showed that shared environmental influences were greater than genetic influences for a low-income group compared to middle- and high-income groups in grade one reading. In contrast, Friend et al.^1^ examined 545 identical and fraternal twins with at least one member of the pair who had reading disability. They reported a G × E interaction and found that genetic influence was higher and environmental influence was lower among children whose parents had a high level of education. The heritability of low reading ability was significantly higher among children whose parents had higher levels of education, indicating that parental education moderated genetic influences on reading disability. Friend et al.^12^ explored identical and fraternal twins with typically developing reading abilities from the US and UK and reported that the heritability of high reading ability increased significantly with lower levels of parental education in both samples. Children whose parents had lower levels of education tend to have stronger genetic influence on their high reading ability. However, in a similarly aged sample, no moderating effects of parental education on genetic influences were found^44^. In addition, brain-behavior relationships critical for reading development are more pronounced in low SES environments^45^.

Overall, the findings from G × E interactions on reading ability are mixed. Much of the research on these topics used a behavioral genetics approach rather than a molecular genetics approach so that the G × E interaction research is mainly limited to twin studies. Further research is necessary to understand how genes interact with environment to affect reading ability. Ideally, a study testing a moderating effect would use a molecular genetics approach predicted on genetic variants previously associated with reading performance and independently replicated.

**Fig. 1:**
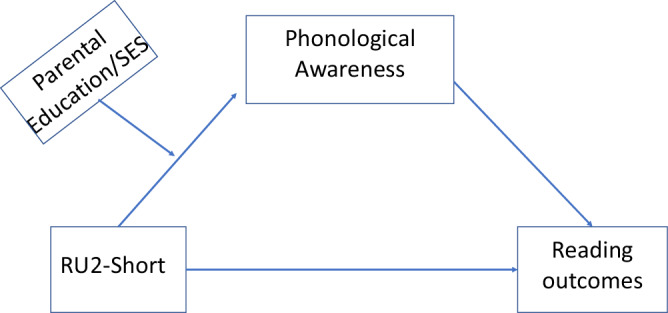
The proposed moderated mediation model. The relationship between RU2-Short and reading outcomes was mediated by the phonological awareness. And this relationship was moderated by different values of parental education and SES.

In the present study, we hypothesize that (1) phonological processing skill mediates the relationship between READ1 and reading outcomes including word reading and comprehension; and (2) that environmental factors moderate the mediation effect of phonological processing skill (see Fig. 1). To test these hypotheses, we analyzed data from the Genes, Reading and Dyslexia (GRaD) Study of 1419 Hispanic-American and African-American participants, ages 8 years to 15 years. For phonological processing skills we used the Elision and Blending subtests of the Comprehensive Test of Phonological Processing (CTOPP)^46^. For reading outcomes we used the Woodcock–Johnson III - Letter-Word Identification and Word Attack^47^ to assess word reading accuracy, Test of Word Reading Efficiency - Sight Word Efficiency and Phonetic Decoding Efficiency (TOWRE)^48^ to assess word reading fluency, and the Standardized Reading Inventory - Passage Comprehension (SRI)^49^ to assess reading comprehension. To assess the home environment, we used responses from the parental questionnaire. All subjects were genotyped for the READ1 allele, which were partitioned into three functional groups (see Methods). For the analysis, we tested a mediation model in which the relationship between READ1 genotype and reading outcomes was explained by phonological processing skills. Next, we investigated a moderation model in which the home environment factors moderated the relationship between READ1 and reading outcomes. Finally, we integrated the moderator into the mediation model and tested the moderated mediation model in which the strength of indirect (mediation) effect was conditional on the value of moderator (home environment factors).

## Results

Means, standard deviations, and overall correlations of all variables are reported in Table 1. The RU2-Short allele was negatively correlated with phonological awareness (*r* = −0.09, *p* < 0.01) and reading comprehension (*r* = −0.08, *p* < 0.01), and was positively correlated with SES (*r* = 0.08, *p* < 0.01). Phonological awareness was significantly correlated with two home environment factors: parental education (*r* = 0.17, *p* < 0.01) and SES (*r* = −0.13, *p* < 0.01) as well as three reading outcomes: word reading accuracy (*r* = 0.68, *p* < 0.01), word reading fluency (*r* = 0.59, *p* < 0.01), and reading comprehension (*r* = 0.56, *p* < 0.01). The three reading outcomes were also positively associated with each other: *r* = 0.84, *p* < 0.01 for the correlation between word reading accuracy and fluency, *r* = 0.68, *p* < 0.01 for the correlation between word reading accuracy and reading comprehension, and *r* = 0.62, *p* < 0.01 for the correlation between word reading fluency and reading comprehension.

**Table 1:** Descriptive statistics and variable intercorrelations

	Mean/SD	1	2	3	4	5	6	7
1. RU2-Short	0.38/0.49	–						
2. Phonological awareness	92.85/14.35	−0.09**	–					
3. Parental education	13.60/2.92	0.05	0.17**	–				
4. Socioeconomic status	0.51/0.50	0.08**	−0.13**	−0.39**	–			
5. Word reading accuracy	94.50/13.75	−0.04	0.68**	0.16**	−0.14**	–		
6. Word reading fluency	92.65/16.75	−0.05	0.59**	0.09**	−0.08**	0.84**	–	
7. Reading comprehension	7.47/3.93	−0.08**	0.56**	0.19**	−0.18**	0.68**	0.62**	–

Phonological Awareness = A composite score of CTOPP Elision and Blending; Word Reading Accuracy = A composite score of WJ-III Letter-Word Identification and Word Attack; Word Reading Fluency = A composite score of TOWRE Sight Word Efficiency and Phonemic Decoding Efficiency; Reading Comprehension = Standardized Reading Inventory.

*SD* Standard Deviation.

**p < 0.01.

The overall correlations among RU2-Short, moderators, i.e., parent education and SES, and reading outcomes tended to be small though significant. Merged moderator levels would potentially also yield small correlations so we independently correlated different levels of parent education moderators: low, medium, and high. Supplementary Table 1 shows the correlations within the low and medium levels of parent education below the diagonal and high level of parent education above the diagonal. There was a small, but statistically significant negative relationship between RU2-Short and reading variables for low and medium levels of parent education. However, this trend was not observed with a high level of parent education in conjunction with reading variables. A small and nonsignificant relationship was found between RU2-Short and reading variables for high level of parental education. Similarly, Supplementary Table 2 has correlations within the low level of SES below the diagonal and high level of SES above the diagonal. Since the SES variable was binary (1 for low level of SES and 0 for high level of SES), we could not compute the correlation between SES and other variables. However, there was a small, but statistically significant negative relationship between RU2-Short and reading variables for low SES. We also found a small but nonsignificant relationship between RU2-Short and reading variables for high SES.

### Tests of mediation

Next, we tested for mediating effects of phonological awareness with the three reading outcomes (see Table 2): (1) word reading accuracy assessed by a composite score of WJ-III Letter-Word Identification and Word Attack, (2) word reading fluency assessed by a composite score of TOWRE Sight Word Efficiency and Phonemic Decoding Efficiency, and (3) reading comprehension assessed by SRI Passage Comprehension. Phonological awareness and RU2-Short accounted for 69% of the variance when word reading accuracy was the dependent variable and RU2-Short was the independent variable. RU2-Short had a significant indirect effect (*b* = −1.74) on word reading accuracy through phonological awareness, with a bootstrapped 95% CI that did not cross zero around the indirect effect (−2.78, −0.68), indicating that phonological awareness had a significant mediation effect. When word reading fluency was the dependent variable, phonological awareness and RU2-Short accounted for 59% of the variance. RU2-Short had a significant indirect effect (*b* = −1.87) on reading fluency through phonological awareness with a bootstrapped 95% CI around the indirect effect (−2.94, −0.74) that did not cross zero, indicating that phonological awareness had a significant mediation effect. When reading comprehension was the dependent variable, phonological awareness and RU2-Short accounted for 56% of the variance. RU2-Short had a significant indirect effect (*b* = −0.40) on reading comprehension with a bootstrapped 95% CI around the indirect effect (−0.64, −0.16) that did not cross zero, indicating that phonological awareness had a significant mediation effect. These analyses showed that phonological awareness mediated the effect of RU2-Short and all three of the reading outcomes.

**Table 2:** Regression results for mediation effects of phonological awareness between DCDC2-READ1 and reading outcomes

	Phonological awareness			Mediator phonological awareness			Phonological awareness		
RU2-short	b	t	CI	b	t	CI	b	t	CI
	−2.64**	−3.30	[−4.20, −1.07]	−2.69**	−3.38	[−4.26, −1.13]	−2.65**	−3.32	[−4.21, −1.08]
	Word reading accuracy			Dependent variables word reading accuracy			Reading comprehension		
RU2-short	0.48	0.86	[−0.62, 1.59]	0.12	0.16	[−1.37, 1.62]	−0.26	−1.40	[−0.61, 0.10]
Phonological awareness	0.66**	34.58	[0.63, 0.70]	0.69**	26.76	[0.64, 0.74]	0.15**	24.32	[0.14, 0.16]
Indirect effect									
Phonological awareness	−1.74		[−2.78, −0.68]	−1.87		[−2.94, −0.74]	−0.40		[−0.64, −0.16]

Phonological awareness = A composite score of CTOPP Elision and Blending; Word reading accuracy = A composite score of WJ-III letter-word identification and word attack; Word reading fluency = A composite score of TOWRE sight word efficiency and phonemic decoding efficiency; Reading comprehension = Standardized reading inventory.

**p < .01.

### Tests of moderation

Next, we tested for moderation effects from parental education or SES (Table 3). Cross-product terms between parental education and word reading accuracy (*b* = 0.92, *p* < 0.01), parental education and word reading fluency (*b* = 1.00, *p* < 0.01), and between parental education and reading comprehension (*b* = 0.22, *p* = 0.01) were all significant. Cross-product terms between SES and word reading accuracy (*b* = −3.52, *p* = 0.02), and between SES and reading comprehension (*b* = −1.05, *p* = 0.01) were also significant.

**Table 3:** Regression results for moderation effects of parent education and SES between DCDC2-READ1 and reading outcomes

Predictor	*b*	*SE*	*t*	*p*
Word reading accuracy				
RU2Short	−14.19	3.97	−3.58	0.00
Parental education	0.50	0.16	3.14	0.00
RU2Short × parental education	0.92	0.28	3.24	0.00
Word reading fluency				
RU2Short	−15.68	4.89	−3.21	0.00
Parental education	0.27	0.19	1.37	0.17
RU2Short × parental education	1.00	0.35	2.85	0.00
Reading comprehension				
RU2Short	−3.67	1.13	−3.25	0.00
Parental education	0.21	0.05	4.77	0.00
RU2Short × parental education	0.22	0.08	2.67	0.01
Word reading accuracy				
RU2Short	0.91	1.11	0.82	0.41
SES	−2.43	0.94	−2.59	0.01
RU2Short × SES	−3.52	1.53	−2.30	0.02
Word reading fluency				
RU2Short	0.32	1.36	0.23	0.82
SES	−1.66	1.15	−1.44	0.15
RU2Short × SES	−3.52	1.87	−1.88	0.06
Reading comprehension				
RU2Short	0.02	0.31	0.07	0.95
SES	−0.99	0.26	−3.76	0.00
RU2Short × SES	−1.05	0.43	−2.45	0.01

Word reading accuracy = A composite score of WJ-III letter-word identification and word attack; Word reading fluency = A composite score of TOWRE sight word efficiency and phonemic decoding efficiency; Reading comprehension = Standardized reading inventory.

*SES* Socioeconomic Status.

### Tests of moderated mediation

To examine whether the strength of the indirect mediation effect of RU2Short was conditional on the value of the either parental education or SES moderators, we then tested a moderated mediation model (Table 4). When word reading accuracy was the outcome, RU2-Short, phonological awareness, and parent education accounted for 69% of the variance, F = 561.13; RU2-Short, phonological awareness, and SES accounted for 68% of the variance, F = 600.41. The interaction terms between RU2Short and moderators (parental education and SES) on phonological awareness were significant (*a* path: *b* = 0.76, 95% CI [.18, 1.35]; *b* = −4.31, 95% CI [−7.42, −1.20]). Additionally, phonological awareness had a significant effect on word reading accuracy (*b* path: *b* = 33.42, 95% CI [0.62, 0.69]; *b* = 34.58, 95% CI [0.63, 0.70]). Although RU2short did not have a direct effect on word reading accuracy, there were conditional indirect effects of three values of parental education (high, medium, low) and two values of SES (high and low) through phonological awareness. The three values of parental education were the mean and plus/minus one SD from the mean (Mean = 13 years, SD = 3 years). There were 402, 428, and 465 participants in high, medium, and low parental education groups, respectively. Our post-hoc division of parental education indicates that there was a negative and nonsignificant correlation between PA and low parental education (r = −0.04, CI = −0.92, 0.33) and a positive and nonsignificant correlation between PA and medium parental education (r = 0.09, CI = −0.21, 5.52), and a positive and significant correlation between PA and high parental education (r = 0.11 CI = 0.11, 3.08). The two values of SES were high (not receiving government assistance) and low (receiving government assistance). There were 701 participants in the high SES group and 708 participants in the low SES group. When parental education was low and medium, there was a significant indirect effect of RU2Short on word reading accuracy through phonological awareness (*c’* path: *b* = −3.39, 95% CI [−5.01, −1.78]; *b* = −1.93, 95% CI [−3.01, −0.86]) but not when parent education was high (*c’* path: *b* = −0.47, 95% CI [−2.01, 1.03]). When SES was low, there was a significant indirect effect of RU2short on word reading accuracy through phonological awareness (*c’* path: *b* = −2.91, 95% CI [−4.36, −1.54]), but not when SES was high (*c’* path: *b* = −0.05, 95% CI [−1.53, 1.45]).

**Table 4:** Regression results for moderated mediation effects of phonological awareness and home environment between DCDC2-READ1 and reading outcomes

Phonological awareness				Mediator			Phonological awareness		
				Phonological awareness					
	b	t	CI	b	t	CI	b	t	CI
RU2-Short	−13.33**	−3.20	[−21.51, −5.15]	−13.52**	4.17	[−21.70, −5.34]	−13.49**	−3.24	[−21.66, −5.31]
PE	0.67**	4.04	[0.34, 0.99]	0.67**	0.17	[0.35, 1.00]	0.67**	4.05	[0.35, 1.00]
RU2-Short x PE	0.76**	2.56	[0.18, 1.35]	0.77**	0.30	[0.19, 1.36]	0.77**	2.60	[0.19, 1.36]
Dependent variables									
Word reading accuracy				Word reading fluency			Reading comprehension		
RU2-Short	0.36	0.62	[−0.78, 1.50]	0.04	0.05	[−1.52, 1.59]	−0.25	−1.31	[−0.63, 0.13]
PA	0.66**	33.42	[0.62, 0.69]	0.68**	25.35	[0.63, 0.73]	0.15**	23.35	[0.14, 0.16]
Conditional indirect effect									
Low PE	−3.39		[−5.01, −1.78]	−3.56		[−5.25, −1.84]	−0.79		[−1.18, −0.42]
Medium PE	−1.93		[−3.01, −0.86]	−2.04		[−3.07, −0.91]	−0.45		[−0.70, −0.20]
High PE	−.47		[−2.01, 1.03]	−0.51		[−2.17, 1.07]	−0.11		[−0.49, 0.26]
Mediator									
Phonological awareness				Phonological awareness			Phonological awareness		
	b	t	CI	b	t	CI	b	t	CI
RU2-Short	−0.08	−0.07	[−2.33, −2.18]	−0.08	−0.07	[−2.33, −2.18]	−0.08	−0.07	[−2.33, 2.18]
SES	−2.12*	−2.18	[−4.04, −.21]	−2.08*	−2.14	[−3.99, −.17]	−2.09*	−2.14	[−3.99, −0.18]
RU2-Short x SES	−4.31**	−2.72	[−7.42, −1.20]	−4.41**	−2.78	[−7.52, −1.30]	−4.34**	−2.74	[−7.45, −1.23]
Dependent variables									
Word reading accuracy				Word reading fluency			Reading comprehension		
RU2-Short	0.48	0.86	[−0.62, 1.59]	0.12	0.16	[−1.37, 1.62]	−0.26	−1.40	[−0.61, 0.10]
PA	0.66**	34.58	[0.63, 0.70]	0.69**	26.76	[0.64, 0.74]	0.15**	24.32	[0.14, 0.16]
Conditional indirect effect									
High SES	−0.05		[−1.53, 1.45]	−0.05		[−1.65, 1.45]	−0.01		[−0.36, 0.33]
Low SES	−2.91		[−4.36, −1.54]	−3.11		[−4.64, −1.61]	−0.67		[−0.99, −0.35]

Phonological awareness = A composite score of CTOPP Elision and Blending; Word reading accuracy = A composite score of WJ-III letter-word identification and word attack; Word reading fluency = A composite score of TOWRE sight word efficiency and phonemic decoding efficiency; Reading comprehension = Standardized reading inventory.

*PE* Parental Education, *SES* Socioeconomic Status.

**p < 0.01; *p < 0 .05.

When word reading fluency was the outcome, RU2-Short, phonological awareness, and parent education accounted for 58% of the variance, F = 323.77; RU2-Short, phonological awareness, and SES accounted for 59% of the variance, F = 360.58. The interaction terms between RU2Short and moderators on phonological awareness were significant (*a* path: *b* = 0.77, 95% CI [0.19, 1.36]; *b* = −4.41, 95% CI [−7.52, −1.30]). Additionally, phonological awareness had a significant effect on word reading fluency (*b* path: *b* = 25.35, 95% CI [0.63, 0.73]; *b* = 26.76, 95% CI [0.64, 0.74]). Although RU2short did not have a significant effect on word reading fluency, there were conditional indirect effects of RU2short on word reading fluency at three values of parental education and at two values of SES through phonological awareness. When parental education was low or medium, there was an indirect effect of RU2Short on word reading fluency through phonological awareness (*c’* path: *b* = −3.56, 95% CI [−5.25, −1.84]; *b* = −2.04, 95% CI [−3.07, −0.91]), but not when parent education was high (*c’* path: *b* = −0.51, 95% CI [−2.17, 1.07]). When SES was low, there was an indirect effect of RU2Short on word reading fluency through phonological awareness (*c’* path: *b* = −0.67, 95% CI [−4.64, −1.61]), but not when SES was high (*c’* path: *b* = −0.05, 95% CI [−1.65, 1.45]).

When reading comprehension was the outcome, RU2-Short, phonological awareness, and parent education accounted for 55% of the variance, F = 278.27; RU2-Short, phonological awareness, and SES accounted for 56% of the variance, F = 302.27. The interaction terms between RU2Short and moderators on phonological awareness were significant (*a* path: *b* = 0.77, 95% CI [0.19, 1.36]; *b* = −4.34, 95% CI [−7.45, −1.23]). Additionally, phonological awareness had a significant effect on word reading accuracy (*b* path: *b* = 23.35, 95% CI [0.14, 0.16]; *b* = 24.32, 95% CI [0.14, 0.16]). Although RU2Short did not have a direct and significant effect on reading comprehension, there were conditional indirect effects of RU2short on reading comprehension at three values of parental education and two values of SES through phonological awareness. When parental education was low or medium, there was an indirect effect of RU2Short on reading comprehension through phonological awareness (*c’* path: *b* = −0.79, 95% CI [−1.18, −0.42]; *b* = −0.45, 95% CI [−0.70, −0.20]), but not when parent education was high (*b* = −0.11, 95% CI [−0.49, 0.26]). When SES was low, there was an indirect effect of RU2Short on reading comprehension through phonological awareness (*c’* path: *b* = −0.67, 95% CI [−0.99, −0.35]) but not when SES was high (*b* = −0.01, 95% CI [−0.36, −0.33]).

## Discussion

In a study of mediation and moderation factors in 1419 African-American and Hispanic-American children, we examined the influence of the genetic variant RU2-Short on word reading accuracy, word reading fluency, and reading comprehension. The results support a moderated mediation model, showing an indirect effect between RU2-Short and reading outcomes through phonological awareness, which was contingent on levels of parental education and SES.

Although the heritability estimates for reading performance are not uniformly high, the variability in the results from previous studies may be partially explained by an indirect relationship between genetic factors and reading^50^. Longitudinal and intervention studies have shown that phonological awareness causally predicts reading outcomes^2–5^. Our results confirm the fully mediating role of phonological awareness in the connection between at least one risk gene (*DCDC2*) and the three reading outcomes that we tested. Other factors that likely contribute to the variability between studies include the small size of the cohorts, differences in assessments, and the study methods (for example, twins versus kinships)^1,7,12^.

The nature of the interaction between genetic variants and environment on reading performance is generallyunder-studied. Consistent with previous studies^20,43^, we show significant interactions between a well-known genetic risk variant (READ1) and home environment on reading outcomes, confirming that the relationship between genetics and phonological awareness can be adjusted by home environment factors. The strength of the indirect effect between risk genes and reading outcomes is conditional on the value of the home environment factors. When parental education and SES were low, there was a strong relationship between RU2-Short and reading performance. This supports the *diathesis-stress model*^51,52^, in which the heritability for reading is greater in a high-stress environment where stressors may lead to expression of risk genes. In contrast to the findings of Friend et al.^1,12^, we do not show that the genetic influence on reading disability is higher among children whose parents have a high level of education; this may be because Friend et al. did their studies in monozygotic and dizygotic twins, whereas we studied unrelated children.

A moderated mediation model could show that the effect of RU2-Short on reading outcomes is transmitted by phonological awareness, varying as a function of parental education and SES. In other words, phonological awareness mediates the relationship between *DCDC2* and reading outcomes when parents have low education level but not when parents have medium and high education levels, and when SES is low but less or perhaps not at all when SES is high. The connection between *DCDC2* and reading performance is indirect through phonological awareness and is adjusted by different values of parental education and SES. While acknowledging the important role of positive home environment, cognitive capabilities (e.g., phonological awareness) of children should be supported to improve their reading achievement more effectively. Both cognitive and environmental factors need to be considered when examining the influence of risk genes on reading.

The present study broadens the scope of gene effects and presents a complex picture of how genes influence reading performance by considering the mediating role of phonological awareness, varying by parental education and SES. The finding is important because it suggests that despite a strong relationship between genes and phonological awareness, which in turn affects reading performance, the linkage between genes and phonological awareness is diminished when home environment is positive, and only becomes strong in more stressful environments.

While reading ability continues to be a critical component of academic success, our results have several implications for education. The findings highlight the importance of phonological processing skill – particularly phonological awareness – as the main factor to explain the connection between genes and reading ability. In the classroom, teachers should continue to target phonics training to enhance reading performance. Results from the present study of gene-by-environment interactions support the idea that risk genes tend to affect reading ability among children with parents having low education and in low SES families. Therefore, strategies to improve educational and home environments could be especially fruitful for children who carry risk genes for reading.

Although our study is limited by its cross-sectional nature and a longitudinal design would have been more appropriate to test for mediation effects, it helps build the theoretical model of the moderated mediation. Furthermore, viewed as a mediator, phonological awareness has been shown to be a causal factor of reading outcomes in both longitudinal and experimental designs^2,5,16^, making the mediation effect viable. Still, future research should examine and replicate our model with longitudinal and intervention data. In addition, we examined the contribution of only a single genetic risk variant, RU2-Short, to reading outcomes. Although RU2-Short is a functional genetic variant in the READ1 regulatory element for *DCDC2* and has been independently replicated, the correlations between it and all the reading variables are small in magnitude though significant. Other genetic risk variants should also be investigated in future studies. Furthermore, our study focuses on two demographic groups (African-Americans and Hispanic-Americans) which have historically been under-represented in genetics research within North America. It is important to note that the findings may not be readily generalizable to all countries such as Scandinavian countries where learning opportunities are not highly related to SES. Moreover, while our study included Hispanic-American participants, we did not assess their proficiency in Spanish due to the primary focus of English reading (dis)ability. To enhance the generalizability of our findings, it will be necessary for future research to explore more diverse populations, encompass bilingualism, consider different contexts, and involve larger cohorts, and expand to additional genes associated with reading.

## Methods

### Participants

There were 1419 self-identified African-American and Hispanic-American children and adolescents who participated in this study. Their age range was from 8 to 15 years. Of the participants, 16% were ages 8–9, 30.5% were ages 10–11, 27.2% were ages 12–13, and 26.3% were ages 14–15. This study was part of a larger, multi-site US and Canadian collaborative Genes, Reading, and Dyslexia (GRaD) project led by Yale University. The full set of sites included Albuquerque, NM; Baltimore, MD; Boston, MA; Boulder and Denver, CO; New Haven, CT; San Juan, PR; and Toronto, Canada. Participants with significant cognitive delays, behavioral problems, emotional/psychiatric disturbances, chronic neurologic conditions, and documented vision or hearing impairment were excluded. Participants were provided with written informed consent to take part in the study. This study was approved by the Human Investigation Committee of Yale University and all the review boards of participating data collection sites.

### Measures

#### Phonological awareness

Phonological awareness was assessed using the Elision and Blending subtests of the Comprehensive Test of Phonological Processing (CTOPP)^46^. In the Blending subtest, phonological segments were synthesized to form a word. In the Elision task, a specified phonological segment was removed from a word, which formed a new word. The score for each subtest represented the number of correct items, converted to a standard score based on age norms. The maximum score was 133 and range was 85. A composite score of both subtests was used to assess phonological awareness in the study.

### Woodcock–Johnson tests of achievement, third edition (WJ-III)

Measures from the WJ-III^47^ included the Letter-Word Identification and Word Attack. subtests. These measures were used to assess word reading accuracy. The WJ-III Letter Word Identification subtest asked the participant to read a list of increasingly complex single English words aloud. The Word Attack subtest required the participant to use knowledge of English phonology to decode a list of increasingly complex non-words or pseudowords in isolation. The total score for each subtest was the number of words read correctly. The maximum score was 133 and range was 110. The standard score based upon age norms was then converted from the raw score. A composite score of both subtests was used to assess word reading accuracy in the study.

### Test of word reading efficiency (TOWRE)

The TOWRE^48^ was a timed measure used to assess word reading fluency. It included subtests of single word reading (Sight Word Efficiency) and single pseudoword decoding (Phonemic Decoding Efficiency). In the subtest of Sight Word Efficiency, the participant was required to read as many words as possible within 45 s. In the subtest of Phonemic Decoding Efficiency, the participant was required to read as many pseudowords as possible within 45 s. The maximum score was 148 and range was 102. Standard scores for each subtest were the number of correctly read words or pseudowords within the time limit, relative to age norms. A composite score of both subtests was used to assess word reading fluency in the study.

### Standardized reading inventory, second edition (SRI)

The SRI^49^ was used to acquire measures of Comprehension and Word Recognition Accuracy. This individually-administered contextual reading test consisted of 10 passages of increasing difficulty, ranging from pre-primer to an eighth-grade level. Accuracy was assessed during oral reading, followed by a series of questions to determine comprehension. Scores were obtained for word recognition accuracy and comprehension on each passage and then converted to standard scores based on age norms. The maximum score was 20 and range was 19.

### Home environment measures

Following consent and assent procedures, parents or guardians completed a questionnaire that covered family background, household resources, and the child’s education and health history. In the questionnaire, we chose two items which best captured home environment based on the literature. One was parents’ or guardians’ reported years of formal education (ranging 6–18 years). The other was the participation in a government assistance program which was used to assess SES. Families that received a government assistance program were coded as 1 and those without receiving such a program were coded as 0.

### Genotyping

Saliva was collected and DNA extracted using Oragene-DNA kits (DNA Genotek) following manufacturer protocols. SNP genotyping for rs2143340 was conducted as part of a larger Illumina HumanOmni2.5-8 bead chip, with genotyping calls screened for quality control measures. The call rate for rs2143340 in the GRaD sample was 0.983.

READ1 genotyping was conducted using PCR amplification and Sanger sequencing at the Yale W.M. Keck DNA Sequencing Facility using standard protocols as previously described^30^. Primer sequences and amplification protocol were as previously described^11^. READ1 alleles were called from chromatograms using a custom program written in C++ (Dr. Yong Kong, available upon request). If the calling program identified errors, chromatograms were manually examined and deconvoluted for allele calling. The call rate for READ1 allele genotyping was 0.987.

The 2445 bp microdeletion on 6p22, which encompasses the READ1 allele within breakpoints in intron 2 of *DCDC2*, was genotyped by allele specific PCR and agarose-gel electrophoresis. Primer sequences, amplification protocol, and gel electrophoresis for genotyping were as previously described^11^. The genotyping call rate for the microdeletion was 0.972.

### Functional groups of READ1 alleles

READ1 alleles were assigned to one of three groups: (1) RU1-1 alleles have only one copy of Repeat Unit 1 (RU1-1: alleles 2, 3, 9, 12, 25, 27); (2) RU2-Long alleles have two copies of RU1 and greater than seven copies of Repeat Unit 2 (RU2: alleles 5, 6, 13, 14, 19, 20, 22, 23); (3) RU2-Short is characterized by alleles that have fewer than six copies of Repeat Unit 2 (alleles 4, 10, 16, 21). Since we previously observed associations with RU2-Short in a group of African-American and Hispanic-American adolescents who had poor reading comprehension skills^30^, we primarily investigated the effect of RU2-Short in the current study.

### Procedure

This study was approved by the Human Investigation Committee of Yale University and all the institutional review boards of participating sites. Parental consent forms and child assent were collected before participation.

### Data analyses

Descriptive statistics and correlations were conducted first. We then tested our hypotheses in three steps. First, we examined a mediation model in which the relationship between READ1 genotype and reading outcomes was explained by phonological processing skill. Second, we investigated a moderation model in which home environment factors moderated the relationship between genotype and reading outcomes. Finally, we integrated the moderator into the mediation model and tested the moderated mediation model in which the strength of the indirect (mediation) effect was conditional on the value of moderator (home environment) factors. PROCESS^53^ (macro for SPSS 26) was used to investigate the moderation, mediation, and moderated mediation effects among reading, environmental, and genetic components as described (www.processmacro.org). Based on a set of conceptual and statistical diagrams defined by a model number, the user chooses a model preprogrammed into PROCESS^53^ corresponding to the model the user wants to estimate. The statistical method used in PROCESS^53^ to investigate direct and indirect effects is Ordinary Least Squares (OLS) regression analysis which is a common practice in observed variable path analysis to estimate the parameters of each of the regression equations. The PROCESS estimates all the path coefficients, standard errors, *t-* and *p*-values, confidence intervals, and various other statistics. In the PROCESS^53^, confidence intervals were calculated via bootstrapping for the indirect effect based on 5000 bootstrap samples (the default in PROCESS^53^). The level of confidence for all confidence intervals was set at 95%. Indirect effects were considered significant for mediation or moderation if they did not cross zero within the 95% CI. The statistical significance level was accepted at 95% or above. In each model, we also controlled the potential confounders, i.e., age and gender. However, neither age or gender were significantly correlated with other variables and did not emerge as significant predictors for other variables, so we did not report their results in the following correlation table and regression tables. In addition, the percentage of variation explained was calculated using partial r^2 of the full model.

### Reporting summary

Further information on research design is available in the Nature Research Reporting Summary linked to this article.

### Supplementary information

Supplementary Information

Reporting summary

## Data Availability

Anonymized data will be made available through collaborative agreement with the PI, Dr. Jeffrey R. Gruen, as specified in the informed consent for the GRaD Study. Interested potential collaborators are urged to contact Dr. Gruen at jeffrey.gruen@yale.edu.

## Code availability

All codes used for the analyses are available by request directed to the corresponding author.
