# Supplementary material for: Phonological awareness mediates the relationship between DCDC2 and reading performance with home environment
Source: NPJ Sci Learn. 2024 May 3;9:36. doi: 10.1038/s41539-024-00247-5 (PMC11068914; doi:10.1038/s41539-024-00247-5)
Supplement: Supplementary file 1 — Supplementary Information [file 41539_2024_247_MOESM1_ESM.pdf]

Supplementary Table 1

Intercorrelations for different levels of parent education

	1	2	3	4	5	6	7
1. RU2-Short	--	-.06	-.06	.09*	.00	.00	-.03
2. Phonological Awareness	-.14**	--	.18**	-.18**	.68**	.56**	.58**
3. Parental Education	.14**	-.04	--	-.38**	.20**	.17**	.23**
4. Socioeconomic Status	.08	-.00	-.10*	--	-.19**	-.14**	-.19**
5. Word Reading Accuracy	-.12**	.68**	-.05	-.03	--	.82**	.80**
6. Word Reading Fluency	-.12**	.61**	-.12**	.02	.87**	--	.75**
7. Reading Comprehension	-.12**	.59**	-.03	-.06	.68**	.77**	--

Note. Phonological Awareness = A composite score of CTOPP Elision and Blending; Word Reading Accuracy = A composite score of WJ-III Letter-Word Identification and Word Attack; Word Reading Fluency = A composite score of TOWRE Sight Word Efficiency and Phonemic Decoding Efficiency; Reading Comprehension = Standardized Reading Inventory. ** $p < .01$. Correlations within the low and medium levels of parent education are below diagonal and high level of parent education are above diagonal.

Supplementary Table 2

Intercorrelations for different levels of SES

	1	2	3	4	5	6	7
1. RU2-Short	--	-.00	.03	-	.03	.01	.02
2. Phonological Awareness	-.16**	--	.24**	-	.66**	.57**	.57**
3. Parental Education	.13**	.03	--	-	.23**	.19**	.23**
4. Socioeconomic Status	-	-	-	--	-	-	-
5. Word Reading Accuracy	-.09*	.69**	.00	-	--	.84**	.80**
6. Word Reading Fluency	-.09*	.61**	-.05	-	.85**	--	.75**
7. Reading Comprehension	-.12**	.61**	.01	-	.77**	.77**	--

Note. Phonological Awareness = A composite score of CTOPP Elision and Blending; Word Reading Accuracy = A composite score of WJ-III Letter-Word Identification and Word Attack; Word Reading Fluency = A composite score of TOWRE Sight Word Efficiency and Phonemic Decoding Efficiency; Reading Comprehension = Standardized Reading Inventory. ** $p < .01$, * $p < .05$
Correlations within the low level of SES are below diagonal and high level of SES are above diagonal.
- Cannot be computed because SES variable is constant (either 0 or 1).
